# Community Engagement and Collaboration between Researchers and Community Stakeholders for Schistosomiasis and Malaria Projects in Ingwavuma, uMkhanyakude District, KwaZulu-Natal

**DOI:** 10.3390/tropicalmed9100236

**Published:** 2024-10-11

**Authors:** Zinhle Mthembu, Moses John Chimbari

**Affiliations:** 1 Department of Anthropology and Development Studies, University of Zululand, KwaDlangezwa 3886, South Africa; 2 Department of Behavioural Science, Medical and Health Sciences, Great Zimbabwe University, Masvingo P.O. Box 1235, Zimbabwe; mjchimbari@gzu.ac.zw

**Keywords:** community engagement, collaboration, KEP researchers, headmen, community advisory boards

## Abstract

Community engagement is a multiphase process that is crucial for successful community-based health interventions. This study investigates the collaborative phase of community engagement, specifically within a co-developed framework implemented in uMkhanyakude District, South Africa. A qualitative case study approach was employed to explore the experiences of key community stakeholders during the collaborative phase of project implementation. Data collection involved key informant interviews, focus group discussions, and direct observation. The findings demonstrate the potential for effective collaboration among village headmen, community advisory board members, and community research assistants to address local health challenges. Community research assistants played a particularly valuable role in facilitating participatory research and hands-on engagement with researchers. However, several barriers hindered the collaborative process, including demanding work conditions, communication issues regarding compensation, inappropriate behavior from the research team, and culturally insensitive interactions. While community-based participatory research offers a promising collaborative approach for addressing health issues, a careful consideration of local socio-cultural dynamics is essential to avoid misunderstandings and overcome potential barriers. Addressing these challenges is crucial to ensuring that collaborative partnerships effectively empower communities and achieve sustainable improvements in health outcomes.

## 1. Introduction

Schistosomiasis and malaria remain significant global public health challenges, particularly in sub-Saharan Africa, where they impose a substantial burden on vulnerable populations [1]. Efforts to control and eliminate these parasitic diseases frequently encounter complications due to ecological, social, and economic factors that are unique to the affected regions [2]. A multifaceted strategy that integrates community engagement and collaboration as key pillars and extends beyond conventional biomedical strategies is required for effective intervention [3,4]. Researchers and project implementers are increasingly emphasizing participatory techniques to promote the sustainability and effectiveness of schistosomiasis and malaria control activities and acknowledging the vital importance of local knowledge, practices, and ownership [5]. Successful community engagement in health projects, particularly those addressing schistosomiasis and malaria, requires a nuanced understanding of community needs, beliefs, and values [6]. This necessitates moving beyond the reliance on researchers solely addressing community-identified problems, as such an approach can raise ethical concerns, erode trust, and neglect crucial cultural sensitivities. While community ownership is crucial, passive reliance on external expertise can foster docility and apathy, ultimately hindering project success [7]. Active community involvement, from project inception to implementation and evaluation, ensures not only a shared understanding of project goals, but also the integration of invaluable local knowledge, bolstering the effectiveness and impact of interventions [8].

However, forging genuine collaborative partnerships between researchers and rural communities, especially in resource-constrained settings often burdened by schistosomiasis and malaria, presents unique challenges. Projects involving complex methodologies, such as those incorporating ecological conservation strategies for vector control, often demand specialized skills, infrastructure, and significant financial investments [6]. This complexity can create barriers to meaningful community participation, if not carefully addressed through equitable resource sharing, knowledge exchange, and collaborative capacity building [9]. Community-based participatory research (CBPR) offers a valuable framework for navigating these challenges by promoting interdisciplinary collaboration and breaking down traditional disciplinary silos [10]. CBPR has been effectively utilized to address health inequities and empower communities in health promotion initiatives [11]. However, practical limitations, including budgetary constraints, funder-imposed deliverables, and project timelines, can influence the depth and scope of community engagement [12]. Furthermore, concerns persist regarding top–down research methodologies that fail to prioritize genuine community collaboration and involvement, undermining the communities these projects aim to serve [13]. Therefore, successful schistosomiasis and malaria projects must prioritize equitable partnerships, acknowledge community expertise, and navigate practical constraints to ensure sustainable and impactful outcomes.

This paper focuses on community engagement and collaboration within the context of schistosomiasis and malaria projects in Ingwavuma, uMkhanyakude District, KwaZulu-Natal, South Africa. This region is largely rural, characterized by diverse landscapes ranging from coastal plains to mountainous terrain, including several rivers and wetlands, which create suitable environments for disease vectors, and presents a unique setting for exploring the dynamics and outcomes of community engagement [14]. In this region, the climate is subtropical, with warm, humid summers, and mild winters, further contributing to vector proliferation [15]. uMkhanyakude is one of the poorest districts in South Africa with high unemployment and limited access to basic services such as clean water and sanitation [16]. Poverty exacerbates vulnerability to infectious diseases [17]. Both schistosomiasis and malaria are endemic in this region; malaria, in particular, poses a significant threat, with the highest rates in the country [18]. The presence of suitable vector habitats, coupled with socio-economic vulnerabilities, contributes to high transmission rates, particularly in communities with limited access to healthcare and preventative measures [16]. HIV prevalence is also high in the district, further complicating health challenges and potentially increasing susceptibility to other infections [18]. 

By examining the collaborative efforts of researchers and community stakeholders, we aimed to understand how community engagement influences project effectiveness, sustainability, and community health outcomes. This paper presents the experiences of community stakeholders who partnered with the KwaZulu-Natal Ecohealth Program (KEP) researchers to plan and execute research projects intended to reduce the burden of schistosomiasis and malaria. Specifically, this study addressed the following key areas: the specific community engagement strategies employed, the roles and responsibilities of different stakeholders, challenges encountered in fostering collaboration, and the impact of community engagement on project outcomes. Through a detailed analysis of the Ingwavuma experience, we seek to provide valuable insights and lessons learned that can inform and strengthen community engagement practices globally in similar contexts. Ultimately, this study contributes to the growing body of knowledge on participatory approaches to disease control and elimination, advocating for community empowerment as a cornerstone of sustainable and equitable health interventions. 

## 2. Materials and Methods

### 2.1. Theoretical Framework 

As shown in Table 1, this paper is based on an CE framework comprised of (i) a community-oriented approach, (ii) a community-based approach, (iii) a community-managed approach, and (iv) a community-owned approach in line with the Community Engagement Vancouver Coastal Health framework “www.vch.ca/ce (accessed on 27 September 2022)”. The stages include (a) informing the community, (b) consulting the community, (c) involving the community, (d) collaborating with the community, and (e) empowering the community.

Using this framework, we actively engaged the community at various levels of participation, ranging from informing, consulting, and involving collaboration and empowerment, ensuring that everyone had a voice and role to play in the project. The Vancouver framework emphasizes the importance of community engagement, highlighting the value of public participation and the right of everyone to have a voice in decisions that impact their healthcare [14].

### 2.2. Study Setting

**Figure 1:**
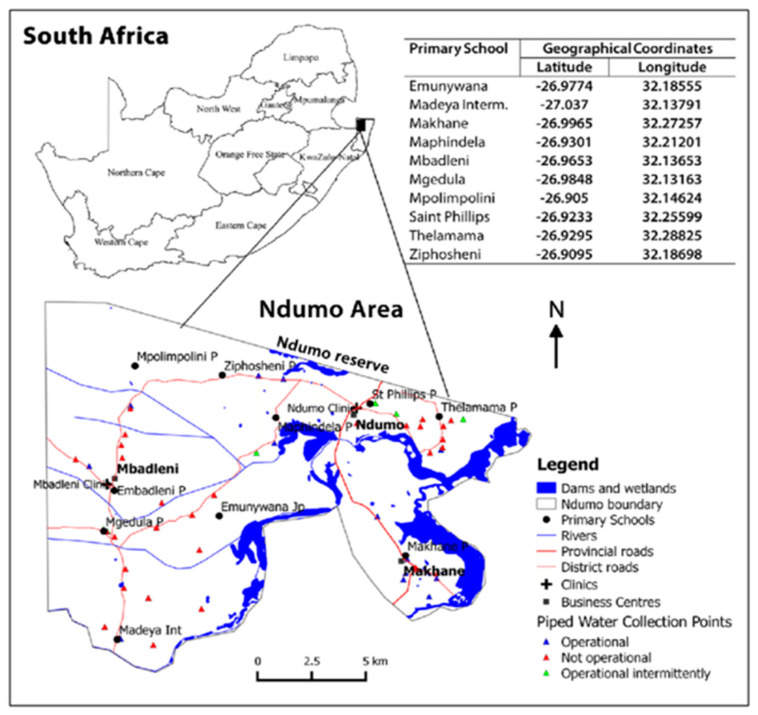
Map showing the MABISA study area in Ndumo, uMkhanyakude. Adapted from [14].

The study was conducted in five villages (Ndumo, Mbadleni, Mgedula, Madeya, and Makhane) in the Ingwavuma area of uMkhanyakude District, the second largest District in the KwaZulu-Natal province, South Africa. This underdeveloped area is near the Ndumo Game Reserve and shares a border with Mozambique and Swaziland. This area has relatively little infrastructure, the road system is currently being developed, and most of the area is only accessible by gravel roads. Schools in villages are widely spaced and provide the barest necessities, with the majority lacking access to running water. The town of Ingwavuma is located in a low-lying area characterized by hot temperatures, still and slowly moving water bodies, and low humidity [15]. Due to its geographic location, the area is vulnerable to vector-borne diseases (VBDs), particularly schistosomiasis (bilharzia) and malaria. This area, which is endemic to many diseases and has poor levels of education, is plagued by poverty [15]. These issues suggest that a broad and inclusive approach to community health education is needed in this area. The study area is served by Mosvold, a district hospital that also oversees two clinics (Mbandleni and Ndumo) that are part of the Jozini Municipality. The five villages involved in MABISA research are depicted on the map (Figure 1) as having numerous primary schools nearby.

### 2.3. The KwaZulu-Natal Ecohealth Program (KEP) Overview

The KwaZulu-Natal Ecohealth Program (KEP) has been working in Ingwavuma since 2014 when the MABISA (Malaria and Bilharzia in Southern Africa) project was initiated. The KEP conducted research projects in uMkhanyakude to lessen the impact of these VBDs. Previously, KEP initiatives in the area included MABISA and TIBA-SA (Tackling Infections to Benefit Africa-South Africa) projects, which started in 2014. These two projects focused on bilharzia and malaria and adopted Ecohealth approaches. The key to the KEP’s success has been the establishment of a governance structure and operations strategy that involves the community to ensure that it fully participates in MABISA/TIBA-SA projects. This was established during the first phase of CE, which is the informing phase [14]. A 12-member community advisory board (CAB) comprising one headman (Induna), two community leaders, three school board members, three community caregivers, and three ordinary community members was established at the inception of the MABISA project and has been functional to date. The headmen (Izinduna) are elected gatekeepers with authority over villages and are accountable to the chiefs, tribal council, and their community [20]. Throughout this paper, the term Ecohealth is used, which commonly involves promoting the health of humans, animals, and ecosystems, including environmental sustainability and socio-economic stability, and conducting research that acknowledges the inextricable connections between the health of all species and their environment [21]. 

### 2.4. Design and Sample

A cross-sectional design embedded with a mixed-methods approach was employed using in-depth interviews (IDIs), focus group discussions (FGDs), and direct observations (DOs) to critically analyze the experiences of community stakeholders in collaborative partnerships on CE processes during the implementation of KEP projects. These three approaches are commonly used and appropriate, particularly in healthcare research [22]. It is advantageous to combine two or more qualitative methods for data collection, which allows complementarity and increases the trustworthiness of the study [23]. A total of 4 FGDs and 34 participants for IDIs, comprising community research assistants (CRAs), a community advisory board (CAB), headmen (who are members of the tribal council), community caregivers (CCGs), which are currently known as community health workers (CHWs), and school principals, as indicated in (Table 2), were purposively selected and interviewed based on their involvement in CE activities and their knowledge about Ecohealth projects implemented in the study area. The existing relationship between KEP and the community of Ingwavuma was used to select the study participants. The authors identified possible important stakeholders using the stakeholder analysis matrix (SAM) method and categorized them into different segments according to their level of interest and influence to achieve the study objectives [24] (see Table 3). The FGDs consisted of 9–12 participants per group, with a mix of genders and different age groups. No incentives or tokens (compensation) were given to the participants. FGDs and IDIs were conducted at the Mosvold Hospital in Ingwavuma. The CAB and CRAs played a crucial role in promoting the concept of community change agents for the prevention and control of vector-borne diseases.

The researcher also used an observational protocol for recording the participants’ information (such as portraits, description of physical settings, and accounts of particular events or activities) while observing. The researcher’s personal thoughts, including “speculation, feelings, issues, ideas, hunches, impressions, and prejudices” were also recorded through reflective notes [25]. Data collection was conducted by an independent researcher with support from the community research assistants. The subsequent community engagement evaluation was led by both authors, with Author 1 being a PhD student under the supervision of Author 2, who also served as the principal investigator for the KEP project. Participants shared their experiences of engagement activities during the implementation of the projects and indicated the opportunities and challenges they encountered. Ethical clearance was granted for this study prior to data collection. Data collection took place between May and December 2021. Written consent was obtained from all the participants. The interviews were conducted in isiZulu and later translated into English by Author 1. For IDIs that lasted between 40–60 min, a semi-structured interview guide was developed. With the participants’ consent, the interviews were digitally recorded and/or notes were written on paper.

### 2.5. Data Analysis

The stakeholder analysis matrix was used to determine the best course of action for each key stakeholder who participated in the study (see Table 3). The recorded interviews were transcribed, the observational field notes were typed, organized, and carefully read (getting a sense of the information and an opportunity to reflect on its overall meaning), and then coded into nodes for analysis using a computer-based software program QSR International Pty Ltd. (Doncaster, Australia), NVivo 12 Pro (version number: NVivo.x64.exe) supplied by Lumivero UK Limited and reseller by Softec based at 14 St George’s road, Claremont, Western Capte, South Africa. As part of Lumivero Inc.’s global sales and marketing activities, Lumivero (London, UK) Ltd. is a subsidiary business of Lumivero Inc. and is set up to market this product in Europe, Middle East and Africa (EMEA). Codes were created and sorted into common themes. The codes were matched with quotes associated with each theme based on similarities, differences, and meanings. Data interpretation was performed using a thematic analysis. This method was adopted to draw conclusions from a combination of data acquired from focus group discussions and in-depth interviews. Four broad themes derived from the thematic analysis are presented in detail in the results section.

## 3. Results

This section presents the findings of a study on stakeholder collaboration in a project. The details of each stakeholder’s role since project inception are available in Table 3. Thematic analysis revealed the following key themes.

### 3.1. Formation of Community Advisory Boards (CAB) and Recruitment of Community Research Assistants (CRAs)

Community advisory boards and community research assistants were established and recruited to prioritize community input in decision making. The collaboration began with the formation of a joint committee responsible for CRA recruitment. The KEP research team emphasized the importance of community representation in all villages during the introduction of the 2014 MABISA project. The CAB serves as a liaison between researchers and the community, facilitating the periodic evaluation of community project perceptions. Twelve members comprise the CAB:

1. Induna (traditional Zulu headman);
2. Community leaders;
3. School board members;
4. Community health workers;
5. Community member representatives (one from each village).

The CAB formation occurred during the initial community meetings. This process is illustrated in the following quote:

“…a committee was formed at the request of the MABISA project team. Initially consisting of four members, the committee expanded to include representatives from Mbandleni, Ndumo, Makhane, and Mgedula as the project’s scope broadened…”

(Induna #3, IDI).

Prior to the community meeting, Induna appointed additional community members, indicating that some community members anticipated selection for committee positions. The function of the CAB was twofold: ensuring ethical conduct within the community and guaranteeing community concerns and interests. One CAB member articulated this role.

“…I wanted to see our community assisted and impacted with knowledge, especially on malaria and bilharzia…Now that CAB members exist, they can transfer the knowledge from the research team to the community…”

(CAB Member 6, FGD).

The importance of CRAs collaborating with the research team for community engagement and research skill development was emphasized by the FGD participant who stated, “It is necessary to have community members in this team”. A team of eight community research assistants was recruited to facilitate community engagement and data collection. This team consisted of seven women and one man, all within the 25–35 age range. Their fluency in IsiZulu was crucial for administering household questionnaires and assisting the research team in collecting data from the participants.

The presence of CABs and CRAs fostered the concept of community change makers for vector-borne disease prevention and control. CRAs collaborated with researchers on data collection, transmission-site identification, and community mobilization. While generally supported by traditional leaders and the community, a misunderstanding arose regarding CRA employment:

“…we were led to believe that the study team was from the Department of Health…and we’d foreseen ourselves becoming millionaires, only to discover that it was not exactly what we had been told…”

(CRA #5, IDI).

Some CRAs had the impression that these roles would be full-time with the Department of Health, offering stable hours and substantial compensation. The reality of part-time, variable-hour engagement with the research team, coupled with the modest compensation, did not align with these expectations, leading to disappointment among some CRAs. This collaborative process began by informing village chiefs and headmen about the study prior to community-wide dissemination.

### 3.2. The Role of Indunas (Headmen/Local Traditional Leadership) in the Collaboration Phase

The initial engagement of the research team with the Mgedula community encountered some challenges. While the tribal council of the Mgedula village approved the study, not all individual villages within the area were fully informed, as illustrated by one Induna’s account:

“…I saw people in the river called Umagwanga and I stop them and ask what they were doing here…they said they were doing research about bilharzia. I then asked them who gave them permission and did they know the king, they said no. I asked them if they knew me, and they said, no. I told them I am a tribal council, and that they should not just show up and head to the river without first consulting us…”

(Induna #2, IDI).

This incident highlights the importance of engaging with local leadership at *every* level, even within communities that have already been granted overall approval. Although community entry was completed at the broader community level, the research team overlooked the specific protocols of individual villages within the Mgedula. This oversight underscores the crucial role of traditional authorities such as indunas in facilitating research.

Following this interaction, the researchers scheduled meetings with all the tribal councils to establish a more collaborative partnership. This experience reinforced the understanding that researchers must inform village headmen in advance of their visits by respecting established protocols. The active participation of local leaders in subsequent research meetings further demonstrates their vital roles. These leaders play a key role in preparing their communities for research activities and ensuring smooth collaboration and community buy-in.

#### The Role of the Community Advisory Board

The community advisory board played a crucial role in bridging the gap between the research team and the community. While the CAB members received meeting allowances and a limited expense coverage rather than salaries, their contributions were significant and well documented. One induna explained the following:

“…since the study team does not reside permanently in our community, it is our responsibility to mediate between the research team and community members…”

(Induna #2, IDI).

The CAB’s responsibilities included the dissemination of information, specifically, relaying project-related information to the community, explaining project activities, and notifying the residents of upcoming research team visits. This is illustrated by the following quote:

“…the committee’s role is to gather all project-related information and disseminate it to the community. In addition, to ensure people’s well-being, explain what has occurred. Inform the community when the research team will visit their homes and give them assurance that there will be no harm from participating in the projects. We always remind them to cooperate…”

(Induna #3, IDI).

Facilitating the community’s acceptance of the research team, particularly given the language barriers, one CAB member noted the following:

“…how we, as CAB members, present the research team to the community determines whether or not community people would accept or reject them. Even though they do not speak the local language, it is part of our responsibility to ensure they’re acceptable in the community, by motivating and giving clarity on the humanity of the team…”

(CAB Member #3, FGD).

Safeguarding community interests and protecting community members, particularly young women, from potential exploitation by researchers was important. This included investigating any inappropriate behavior and reporting it to the headmen:

“…CAB members are also given the prerogative to investigate the project if there are underlying factors pertaining to the researcher’s behavior. For example, we have young girls in our community, and if the researchers divert from what they came here for but decide to have love affairs with them, we should investigate such behaviors and report them to the headmen. We have observed some projects we once had in our area, whereby individuals come but end-up impregnating girls in the community…”

(CAB Member #6, IDI).

Beyond these responsibilities, the CAB members also gained valuable research skills and knowledge through their participation. Their involvement in project meetings and workshops ensured community representation in the planning and decision-making processes, fostering genuine and transparent collaboration. This collaborative approach allowed the CAB and indunas to understand the research activities and their impact on their communities.

### 3.3. Opportunities of Collaboration

Collaboration between the research team and the community provided numerous opportunities for capacity building and knowledge exchange. These opportunities extend beyond the immediate research goals and foster personal and professional growth within the community. This collaboration enabled some CAB members to attend international workshops, a first-time experience for many. This exposure facilitated valuable networking opportunities and broadened their perspective. One CAB member shared the following:

“…being in collaboration with the research team, I have gained a lot. I have been given the opportunity to visit Zimbabwe. Everything included passports, flight tickets, and accommodation. We trained each day from 7 am to 4 pm. There were presentations, and I also had a chance to present my village, which is something I have never done before. I learned to communicate with people who did not speak my own language. I have made connections in Zimbabwe. It was a wonderful and enlightening experience…”

(CAB member #8, FGD).

The KEP project, with its broader health focus, has provided extensive community training and workshops. These initiatives strengthened collaboration and facilitated the community’s understanding of this research. Community health workers reported significant gains in their knowledge and skills:

“…during the MABISA project, we have also taken to Jozini for training, and the research managers booked us in a beautiful hotel. They taught us about stakeholders, malaria, schistosomiasis transmission, and other research skills. Through KEP projects, I have been exposed to so many things…”

(CHW #4, FGD).

“…In other meetings, the research team taught us about stroke, diabetes, and blood pressure, providing the community with a wealth of knowledge. We can apply the knowledge we learned in our daily work as caregivers…”

(CHW #2, IDI).

The KEP fostered community engagement through bi-annual feedback meetings utilizing edutainment techniques such as drama and poetry performed by the community research assistants, schoolchildren, and youth. These meetings served as a platform for sharing study updates, disseminating findings, and uniting villages. The schools’ involvement in annual performance art competitions further enhanced community health education and research uptake by incorporating indigenous theatre practices and relatable metaphors.

#### Capacity Building and Shifting Community Perceptions

The KEP project invested significantly in capacity building within the community, particularly among the CRAs. This investment not only enhanced research capacity but also fostered trust and shifted community health perceptions. The KEP team provided extensive training to CRAs, covering research ethics, the epidemiology of malaria and schistosomiasis, fundamental research techniques, quality assurance, and data-gathering skills. CRAs also gained practical experience in parasitology and specimen collection. This training had a profound impact on participants:

“…I have benefited a lot since it was MABISA until it changed to TIBA, I am not going to lie. Now I have a lot of experience, which will be useful if I apply to another job. With the knowledge I have learned, I can succeed in various work fields. I received this information for free, and the experience I obtained is valuable to me because I may not have had the funds to pay for it otherwise. Also, receiving a certificate was a bonus…”

(CRA #6, IDI).

Initially, the community viewed the research team with suspicion. However, the consistent presence and community-focused approach of the KEP project fostered trust and led to positive changes in health-seeking behaviors, particularly among men:

“…we always see the research team moving around with scales, weighing children and the elderly, so the community easily notices them when they are not present in the area. Men preferred not to visit clinics, but since the team began working in our community, this changed. This shows that the door-to-door visits that they do have a positive impact on the community…”

(CAB member #8, FGD).

### 3.4. Challenges of Collaboration

While collaboration between the community and researchers yielded significant benefits, some challenges emerged that warrant attention. Community research assistants expressed appreciation for the learning opportunities and skills acquired through this project. These include training in qualitative and quantitative data collection, specimen collection and processing, vector mapping, and basic geospatial techniques. The overall experience and exposure gained through participation in the research activities were highly valued. Despite these positive aspects, CRAs also voiced concerns regarding working hours, communication within the research team, and the format of the certificates of participation. Specifically, they expressed their dissatisfaction as follows:

#### 3.4.1. Poor Working Conditions

While collaboration offered valuable learning experiences, several CRAs expressed concerns about working conditions, particularly regarding work schedules, meal breaks, and compensation. CRAs reported inconsistent working days and a lack of advanced notice regarding fieldwork. This makes it difficult to plan personal commitments and effectively manage their time. One CRA noted the following:

“…the only thing I have observed is that the team is not well organized. However, our working days were inconsistent. They would tell us in the morning that you are needed in the field. It is like the researchers do not plan their work on time and it is not right…”

(CRA #4, IDI).

The absence of consistent meal breaks and provision for lunch or tea was a significant concern. The CRAs highlighted the difficulty of working without adequate breaks and nourishment:

“…working relationships are bad, there is no cooperation at all. There is no job in which you cannot have a lunch break. I am saying this because, if there was cooperation, they would consider that some people do not eat in the morning, and there should be teatime and lunchtime in the workplace. Some of us cannot eat during the early hours of the day. We receive none of these, and we end up conducting research at someone’s home while hungry…”

(CRA #1, IDI).

The researchers explained that the fluctuating nature of research activities necessitated variations in the number of CRAs required on different days. To ensure an equitable distribution of fieldwork opportunities and experiences, a rotation system was implemented. While intended to be fair, this system inadvertently led to a reduction in individual stipends for some CRAs during certain field visits. The research team encountered significant logistical challenges due to the remote location of the study sites and the time-sensitive nature of sample processing. These challenges affected working conditions, particularly with regard to meal breaks and work schedules. The distance of the study sites from the main shopping center made it difficult for the research team to access food, resulting in extended periods without meals. Departing early, before shops opened, further compounded this issue, particularly for CRAs that relied on purchasing food rather than bringing packed lunches. The intense nature of the work, particularly during stool and urine sample collection, often precluded regular meal breaks. The time-sensitive processing of urine samples further limited their flexibility.

While the logistical challenges were undeniable, several potential solutions could have been explored to mitigate their impact on the CRAs: Offering packed lunches or providing meal allowances to CRAs could have addressed the issue of limited food access. Investigating alternative transportation or scheduling options, even if costly, could potentially reduce travel time and allow for more predictable workdays. Consulting with CRAs to understand their needs and collaboratively brainstorm solutions could have fostered a more positive working environment and potentially identified more effective strategies.

#### 3.4.2. Poor Time Management

Time management and communication issues created significant difficulties for the community research assistants, impacting their personal lives and potentially hindering collaborative research relationships. CRAs frequently experienced unpredictable pick-up times, often waiting for extended periods. A lack of advanced notice regarding work schedules disrupted their personal plans and created unnecessary stress.

“…The team leader will tell you that they will pick you up at 7 a.m., and you will wait for an hour or longer…Sometimes you go to bed not knowing what you’re going to work on the next day…”

(CRA #2, FGD).

The absence of defined work hours and frequent late drop-offs, sometimes as late as 8 pm, further complicated matters for the CRAs: “…the knock-off time is not always kept…The number of hours we will be working each day should be specified, and we should adhere to those hours…” (CRA#1, FGD).

While the limited transportation options and long distances undoubtedly contributed to scheduling difficulties, these factors should not overshadow the lack of effective planning and communication. While acquiring a second vehicle improved the situation, it did not fully resolve the underlying issues.

Addressing these challenges requires a multipronged approach focused on improved planning, communication, and respect for the CRAs’ time. The team leader should have established clear work schedules in advance and communicated them promptly to the CRAs; implemented a system for notifying CRAs of any changes to the schedule as soon as possible; and involved CRAs in the planning process to foster a sense of ownership and shared understanding of the logistical constraints. Transparency regarding the reasons behind scheduling decisions is crucial, as is recognizing that CRAs have personal lives and commitments outside of the research project. Consistent and predictable work schedules demonstrate respect for their time and contribute to a more positive and productive working relationship. Where possible, flexible work arrangements to accommodate the CRAs’ needs and minimizing disruptions to their personal schedules should be considered.

#### 3.4.3. Misconduct of the Research Team Leader

The CRAs reported instances of unprofessional behavior by the research team leader, which significantly impacted their working relationship and the overall project. The team leader’s frequent intoxication during work hours led to confusion and misunderstandings regarding stipend payments, creating unnecessary stress and distrust among the CRAs: “…the project’s team leader used to drink a lot and would pay us our stipend when he is drunk…The next day, there would be a misunderstanding over whether or not he had given us extra money…” (CRA #5, IDI).

The team leader’s intoxication during work hours constitutes a serious breach of professional conduct. While the principal investigator’s swift response in terminating the team leader’s employment is commendable, this incident underscores a critical need to address such issues proactively. This case exemplifies the often unacknowledged challenges that can arise in research projects, particularly, the tendency to overlook or suppress problematic behavior, especially within community-engaged research. These findings demonstrate the crucial role of team conduct in ensuring appropriate community engagement. The absence of disciplinary measures and effective monitoring can negatively impact the entire project’s reputation and erode community trust. A single act of misconduct by a researcher, particularly one in a leadership position, can generate significant scrutiny from the community and jeopardize the project’s integrity.

#### 3.4.4. Inadequate Compensation

Concerns arose regarding the perceived inadequacy of CRA compensation, particularly given the rising cost of living and the project’s inability to increase payments as requested. This issue stemmed from a fundamental misunderstanding: CRAs participated on a voluntary basis, receiving a stipend as a token of appreciation rather than a salary for employment. However, initial project communication failed to adequately convey this distinction, leading to a community perception of full-time employment for CRAs.

One CRA stated, “…the R170 allowance…is too little. The community… [perceives this] as a full-time job…and we are getting decent salaries…But this money is not even enough to buy cosmetics…” (CRA #3, IDI).

Initially set at ZAR 150, the stipend was later raised to ZAR 170 after CRA complaints. Typical CRA engagement spanned 3–5 days. While a stipend increase would have been ideal, budgetary constraints posed a significant obstacle. Research projects necessitate pre-approved budgets with stringent auditing against actual expenditures, limiting flexibility for subsequent stipend or salary adjustments. The eventual R150–R170 increase was facilitated by incorporating funds from new, aligned projects undertaken by the principal investigator within the same area. This highlights the inherent challenges in reconciling fixed budgets with evolving economic realities and community expectations in long-term research projects. Further research into alternative compensation models and communication strategies for community-engaged research is warranted.

#### 3.4.5. Undermining CRAs Due to English Incompetence

The CRAs’ limited English proficiency created a significant barrier to communication, hindering their ability to express opinions and potentially contributing to the negative perceptions of their role. This language barrier resulted in feelings of oppression and marginalization among CRAs, who felt unable to voice concerns in fear of ridicule or dismissal. One CRA articulated the following:

“…we struggle to communicate in English…during meetings…We end up keeping our mouths shut…because we are afraid to speak English…” (CRA #8, FGD). Another added, “…we feel oppressed when they expect us to express ourselves in English…we already feel defeated…”

(CRA #1, FGD).

Beyond the immediate communication challenges, this situation reveals a deeper power imbalance. The CRAs’ perceived acquiescence due to language difficulties raises ethical concerns regarding informed consent and the potential for misrepresentation. Their inability to fully understand project details or to raise questions creates a risk of unintentional ethical violations. The researchers’ failure to accommodate the CRAs’ language needs underscores a lack of empathy and highlights the importance of linguistic inclusivity in research. Future projects should prioritize recruiting researchers proficient in the local language or providing skilled interpreters. Direct communication, facilitated by language proficiency or accurate interpretation, is crucial for fostering trust, ensuring informed consent, and accurately capturing community perspectives. Researchers should also consider actively learning the local language to minimize reliance on intermediaries and gain a more nuanced understanding of on-the-ground realities. This can mitigate the risk of miscommunication and ensure that community voices are genuinely heard and respected.

## 4. Discussion

This study investigated community experiences during the collaborative phase of the KEP projects, revealing the importance of establishing and maintaining robust stakeholder partnerships, particularly given the extended project timelines (2014–2022). This aligns with previous research emphasizing the necessity of long-term engagement processes that accommodate diverse perspectives and facilitate consensus-building [26]. Our findings demonstrate sustained collaboration between the research team and community stakeholders, exemplified by the establishment of working groups like the CAB and CRAs, which played integral roles in project decision-making.

Furthermore, the study highlights a strong correlation between collaborative partnerships and mutual benefits. Community involvement in project design, data collection, and dissemination fostered shared goals, reciprocity, and mutual benefit, reflecting a deliberate effort to prioritize community interests. This resonates with the literature advocating for mutuality and reciprocity in community-research partnerships [27]. Effective collaboration necessitates a shared vision and mutually beneficial outcomes.

Our findings also support the distinction between “transactional” and “transformational” partnerships [28]. While transactional partnerships prioritize the exchange of individual or institutional interests, transformational partnerships are characterized by their comprehensive nature, collaborative planning and management, mutual benefit, long-term commitment, strong leadership support, and a focus on community capacity building [28]. The KEP projects, with their extended timeline and community-centric approach, appear to exemplify aspects of transformational partnerships. However, further investigation is needed to fully assess the extent of community capacity building and the long-term impacts of these collaborations. Building upon the previous year’s experiences, the current study underscores the value of sustained engagement and community empowerment in achieving project goals and fostering sustainable positive change within the community. Future research should explore the specific mechanisms through which these long-term partnerships translate into tangible community benefits and investigate the potential challenges and opportunities associated with maintaining such collaborations over extended periods.

This study reveals an evolution in community leadership towards health education facilitated by collaborative partnerships. The impact is evident in increased community knowledge regarding vector-borne diseases (schistosomiasis and malaria), enhanced research skills, and numerous learnings derived from the KEP projects [29]. This echoes findings from a comparable study in Ingwavuma, where limited prior research experience was transformed through collaborative co-learning and engagement opportunities [14]. 

From initial interactions, stakeholder contributions were significant. The study confirms the strategic role of traditional leaders in navigating study approvals and ensuring successful implementation. This aligns with previous research highlighting the effectiveness of collaborating with traditional leaders in fostering community acceptance of research projects [30]. Our findings indicate that community research assistants, headmen, and community advisory board members are recognized as community change-makers in vector-borne disease prevention and control. Traditional leaders are instrumental in ensuring smooth project implementation with full community support. This reinforces existing evidence supporting community engagement through neutral spaces and leadership that fosters collaboration, shared goals, and social transformation [31]. 

Furthermore, the study demonstrates the value of CRAs and CAB members as credible resources and partners in addressing community health issues. Workshops and training using locally adapted communication tools strengthened collaborations and created opportunities for international partnerships. Capacity development initiatives, including numerous training sessions, equipped community members with health knowledge and research skills, enabling them to participate in data collection and promote community-driven change for vector-borne disease prevention and control. The close collaboration between the research team and community leadership, particularly traditional leaders, was crucial for project success. This partnership facilitated community recognition and an acceptance of project activities. Local-level structures were strengthened to support co-designed, community-based projects through genuine collaboration. This study reinforces the potential of community-based participatory research as a collaborative approach integrating systematic investigation, community participation, and action to address health challenges [32]. 

While community trust was fostered through the CAB members and CRAs, when enabling the project to remain informed about community perceptions and maintain socio-cultural relevance [33], several challenges emerged in sustaining these collaborative relationships. One key challenge involved communication barriers between researchers and CRAs. Language differences hindered community members from fully expressing their opinions, echoing the existing literature on the challenges of healthcare provision in multilingual and multicultural settings [34]. Beyond mere miscommunication, participants reported feeling mistreated due to the researchers’ perceived lack of empathy regarding their English proficiency. CRAs felt undervalued and disrespected by researchers who did not attempt to understand them in their own language.

Another significant challenge involved inconsistent work hours and a lack of breaks during data collection. Researchers sought to maximize field visit time, creating tension with CRAs who prioritized standard working hours and felt overworked and potentially abused. An incident involving a team leader arriving at work intoxicated and mistreating CRAs further exacerbated these tensions and created misunderstandings regarding stipend payments. These unresolved issues posed a significant threat to the collaborative relationship. Finally, compensation emerged as a point of contention. While stipends were intended as tokens of appreciation and to cover field-related expenses, CRAs viewed the work as deserving of standard wages. Despite understanding the project’s non-obligatory payment structure, CRAs expressed financial needs that required more substantial compensation. Having forgone other opportunities to participate in the project, and being perceived as employed by the community, CRAs sought greater financial support from the project.

The concerns raised by the CRAs highlight the importance of clear communication, consistent scheduling practices, and adequate provisions for meals and breaks. While the researchers’ intentions to provide equitable opportunities are understandable, exploring alternative approaches to scheduling and compensation could mitigate the negative impacts on CRAs and improve overall working conditions. An open dialogue between researchers and CRAs is crucial for finding mutually beneficial solutions. While acknowledging the constraints posed by the research environment is important, prioritizing the well-being of the research team, including the CRAs, is crucial. Proactively addressing these logistical challenges and exploring potential solutions, even if imperfect, demonstrates respect for the CRAs’ contributions and fosters a more equitable and sustainable research partnership. 

Based on the study’s findings we suggest specific implementation strategies in which sustained collaborations can be implemented. This might include developing clear roles and responsibilities for all stakeholders; establishing regular communication channels and feedback mechanisms; creating a sustainable funding model for long-term activities; empowering local communities to take ownership of control programs; and integrating schistosomiasis and malaria control efforts with other community health initiatives.

## 5. Conclusions

Successful community engagement hinges on collaboration with community stakeholders throughout all project phases. This study demonstrated that establishing a community advisory board and recruiting community research assistants effectively bridged the gap between researchers and the community. This facilitated transparent, democratic, and equitable decision-making processes, ultimately contributing to project completion. The KEP research team’s efforts to involve community members in various project aspects fostered new networks and skill development within the community. Furthermore, consulting local-level leaders and adhering to established community entry protocols, including engagement with traditional authorities, proved crucial for smooth project implementation. Positive collaborative relationships between the research team and the community were fostered by embracing partners such as the CRAs and CABs.

Notwithstanding these successes, several challenges emerged, primarily stemming from researcher conduct. These included extended working hours, inadequate stipends, language barriers, researcher misbehavior, and the undervaluing of the CRAs’ opinions. These challenges threatened the collaborative efforts and risked undermining the gains achieved through community involvement in citizen science activities. This study underscores the critical importance of respecting and valuing community members in collaborative partnerships, highlighting the transformative potential of such values in researcher–community relationships. Based on the study’s findings and the experiences of both researchers and the community, we formulated specific recommendations for future research. These recommendations might include investigating the long-term sustainability of community-led health interventions; developing culturally appropriate health education materials; exploring alternative incentive structures for community research assistants and evaluating the effectiveness of integrated approaches to address multiple parasitic infections.

## Figures and Tables

**Table 1:** Approaches to community engagement. Adapted from the WHO [19].

Level 1	Level 2	Level 3	Level 4
Community-Oriented Approach	Community-Based Approach	Community-Managed Approach	Community-Owned Approach
The community is *informed* and mobilized to participate in addressing immediate short-term concerns with external support.	The community is *consulted* and *involved* to improve access to health services and programs by locating interventions inside the community with external support.	There is *collaboration* with leaders of the community to enable priority settings and decisions from the people themselves with or without external support.	Community assets are fully mobilized, and the community is *empowered* to develop systems for self-governance, establish and set priorities, implement interventions, and develop a sustainable mechanism for health promotion with partners and external support groups as part of a network.

**Table 2:** Distribution of key informants and number of interviews conducted.

Key Informants	In-Depth Interviews (IDIs)	Focus Group Discussions (FGDs)	Total Number of Participants (IDIs and FGDs)
Community Research Assistants (CRAs)	7	1 FGD = 8 participants (1 man, 7 women)	15
Community Advisory Boards (CABs)	6	1 FGD = 8 participants (3 men, 5 women)	14
Headmen	3	0	3
Community Health Workers (CHWs)	2	2 FGDs = 20 participants (6 men, 14 women)	22
School Principals	4	0	4
TOTAL	22	4	58

**Table 3:** Stakeholder analysis matrix.

Stakeholder Name	Impact: How Much Does the Project Impact Them? (Low, Medium, High)	Influence: How Much Influence Do They Have over the Project? (Low, Medium, High)	What Is Important to the Stakeholder?	How Could the Stakeholder Contribute to the Project?	How Could the Stakeholder Block the Project?	Role/Function in Collaboration
Village Headmen	High	High	Maintaining and sustaining collaborative partnerships that have been established with the KEP research team.	Organize community meetings and activities in villages to disseminate schistosomiasis research findings.	Rejecting the study and denying Gatekeeper approval.	Village headmen are the main points of contact for the entire community. They are in charge of all traditional matters pertaining to the health and safety of local people. The headman (Induna), who in turn reports to the great King (Isilo), provides reports to the Chief (Inkosi).
Community Advisory Boards (CABs)	High	High	Gather the community members for meetings and grant researchers’ permission to enter their villages and conduct research.	Informs community members about new health trends and issues in their area by coordinating the dissemination of information to community members through the village headmen.	Discouraging the community from taking part in the KEP research projects.	CRAs are used to mobilize the community members on behalf of the researchers to come and participate in learning activities that can promote health and reduce the burden of the disease among the community members
Community Research Assistants (CRAs)	High	High	Mobilizing community members to participate in the KEP projects.	Assist researchers in collecting data.	Withdrawing from the study.	Collecting data with researchers. Assisting in transferring knowledge on health diseases in the local language.
Community Health Workers (CHWs)	Medium	High	Make the link between the community and the health system. Maximizing quality of care for patients. Supplying equipment and drugs for treating School children found with infections in the schools.	Conduct door-to-door visits in the community to educate the community about health-related infections occurring in the area.	Withdrawing from the study.	CHWs visit patients in their homes to provide support with health difficulties and help home-based patients with medicine. They engaged community members through home visits. Distributing pamphlets to the community on behalf of the KEP.
School Principals	Medium	Medium	Educate schoolchildren and make sure that they are recruited for parasitology research by the KEP.	Mobilizing schoolchildren and making sure that the knowledge is transferred to school learners.	Rejecting the study and denying access to schoolchildren.	School principals are there to help schoolchildren to learn about schistosomiasis in the classroom. Encourages learners to perform screening and treatment for schistosomiasis on a regular basis. Makes sure learners utilize books, booklets, and posters provided by researchers. Schools are great place to host meetings with parents to educate them about schistosomiasis.

## Data Availability

The raw data used and/or analyzed during the current study will be made available from the corresponding author on reasonable request.
